# (1-Ferrocenyl-4,4,4-trifluoro­butane-1,3-dionato-κ^2^ *O*,*O*)bis­(triphenyl­phosphane)copper(I)

**DOI:** 10.1107/S1600536811001796

**Published:** 2011-01-22

**Authors:** Tobias Rüffer, Chris C. Joubert, Blenerhassit E. Buitendach, Jannie C. Swarts, Alexander Jakob, Heinrich Lang

**Affiliations:** a Technische Universität Chemnitz, Fakultät für Naturwissenschaften, Institut für Chemie, Lehrstuhl für Anorganische Chemie, Strasse der Nationen 62, 09111 Chemnitz, Germany; b Department of Chemistry, University of the Free State, PO Box 339, Bloemfontein, South Africa

## Abstract

In the title mononuclear coordination complex, [CuFe(C_5_H_5_)(C_9_H_5_F_3_O_2_)(C_18_H_15_P)_2_], the Cu^I^ ion is coordinated by the chelating β-diketonate 1-ferrocenyl-4,4,4-trifluoro­butane-1,3-dione ligand through two O atoms and the two datively bonded triphenyl­phosphane ligands resulting in a distorted tetra­hedral coordination sphere. The Cu^I^ ion, together with its chelating butane-1,3-dione group, is mutually coplanar [greatest displacement of an atom from this plane = 0.037 (1) Å], and the Cu^I^ ion lies slightly above [0.013 (1) Å] the plane. The overall geometry, including the bond distances and angles within the complex, corresponds to those of other reported copper(I) β-diketon­ates featuring organic groups at the β-diketonate ligand.

## Related literature

For β-diketone chelates in lanthanide coordination chemistry, see: Tsukube *et al.* (2002 ▶); Kaizaki (2006 ▶). For the chemistry of platina-β-diketones, see: Steinborn (2005 ▶). For the structure of Cu(II)-β-diketones, see: Gromilov & Baidina (2004 ▶). For the preparation of volatile Cu(I)-β-diketones and their chemistry, see: Shin *et al.* (1991 ▶); Chi *et al.* (1992 ▶) and for the application of volatile Cu(I)-β-diketones in CVD processes (CVD = chemical vapour deposition), see: Fahlman (2006 ▶); Tiitta & Niinistou (1997 ▶); Chen *et al.* (2001 ▶); Doppelt (1997 ▶). For photoelectron spectroscopy and electronic structure studies of metal-β-diketones, see: Vovna *et al.* (1998 ▶). For the application of Cu(I)-β-diketones in ALD processes (ALD = atomic layer deposition), see: Waechtler *et al.* (2009 ▶). For applications of Cu(I)-hexa­fluoro­acetonates, see: Pampaloni *et al.* (2005 ▶); Doyle *et al.* (1985 ▶). For other copper(I) β-diketonate derivatives, see: Yang *et al.* (2001 ▶); Marchetti *et al.* (2000 ▶); Croxtall *et al.* (2003 ▶); Herberhold *et al.* (2004 ▶). For a related ferrocenyl derivative of the title compound, see: du Plessis *et al.* (1999 ▶).
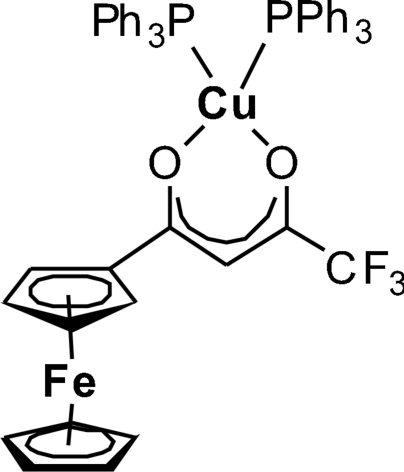


## Experimental

### 

#### Crystal data

[CuFe(C_5_H_5_)(C_9_H_5_F_3_O_2_)(C_18_H_15_P)_2_]
*M* *_r_* = 911.15
Monoclinic, 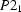

*a* = 11.02755 (13) Å
*b* = 16.8178 (2) Å
*c* = 12.50787 (14) Å
β = 115.2456 (14)°
*V* = 2098.15 (5) Å^3^
*Z* = 2
Mo *K*α radiation
μ = 0.98 mm^−1^
*T* = 120 K
0.56 × 0.48 × 0.18 mm

#### Data collection

Oxford Diffraction Gemini S diffractometer
Absorption correction: multi-scan (*CrysAlis RED*; Oxford Diffraction, 2006 ▶) *T*_min_ = 0.943, *T*_max_ = 1.000
21096 measured reflections
8248 independent reflections
7761 reflections with *I* > 2σ(*I*)
*R*_int_ = 0.015

#### Refinement

*R*[*F* ^2^ > 2σ(*F* ^2^)] = 0.020
*wR*(*F* ^2^) = 0.052
*S* = 1.03
8248 reflections
532 parameters
1 restraint
H-atom parameters constrained
Δρ_max_ = 0.37 e Å^−3^
Δρ_min_ = −0.25 e Å^−3^
Absolute structure: Flack (1983 ▶), 3945 Friedel pairs
Flack parameter: −0.010 (6)

### 

Data collection: *CrysAlis CCD* (Oxford Diffraction, 2006 ▶); cell refinement: *CrysAlis RED* (Oxford Diffraction, 2006 ▶); data reduction: *CrysAlis RED*; program(s) used to solve structure: *SHELXS97* (Sheldrick, 2008 ▶); program(s) used to refine structure: *SHELXL97* (Sheldrick, 2008 ▶); molecular graphics: *ORTEP-3 for Windows* (Farrugia, 1997 ▶); software used to prepare material for publication: *WinGX* (Farrugia, 1999 ▶).

## Supplementary Material

Crystal structure: contains datablocks I, global. DOI: 10.1107/S1600536811001796/si2308sup1.cif

Structure factors: contains datablocks I. DOI: 10.1107/S1600536811001796/si2308Isup2.hkl

Additional supplementary materials:  crystallographic information; 3D view; checkCIF report

**Fig. 2.**
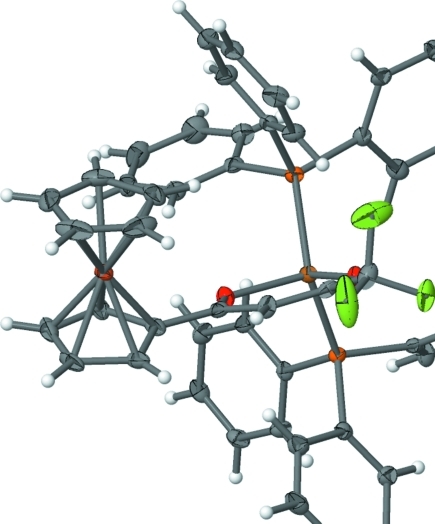
View of the title compound showing displacement ellipsoids at the 50% probability level.

Enhanced figure: interactive version of Fig. 2

## Figures and Tables

**Table 1:** Selected bond lengths (Å)

O1—Cu1	2.0821 (12)
O2—Cu1	2.0783 (11)
Cu1—P2	2.2405 (5)
Cu1—P1	2.2529 (5)

**Fig. 1.**
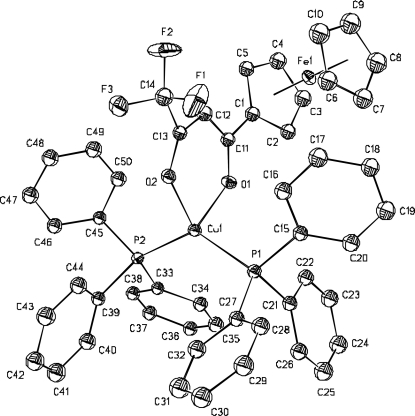
Molecular structure of the title compound. All hydrogen atoms have been omitted. Displacement ellipsoids are drawn at the 50% probability level.

## supplementary crystallographic information

### Comment

Well known coordination compounds in transition metal chemistry are metal β-diketonates and Lewis-base metal β-diketonates, see: Gromilov & Baidina (2004); Steinborn (2005); Tsukube *et al.* (2002); Kaizaki (2006); Vovna *et al.* (1998). A multitude of diverse derivatives have been prepared to date using different synthesis methodologies with copper(I) β-diketonates and Lewis-base copper(I) β-diketonates being one family of compounds. These molecules are, for example, very well suited as volatile CVD (= Chemical Vapor Deposition), see: (Fahlman (2006); Tiitta & Niinistou, (1997); Chen *et al.* (2001); Doppelt (1997), and ALD (= Atomic Layer Deposition), see: Waechtler *et al.* (2009) precursors in the deposition of highly pure and thin copper layers on different substrates, *i. e.* TiN-coated silicon oxide substrate materials.

The title coordination compound is a derivative of the family of transition metal β-diketonates in which the copper(I) center possesses a pseudo-tetrahedral environment set-up by the chelate-bonded organometallic 1-ferrocenyl-4,4,4-trifluorobutane-1,3-dione (see: du Plessis *et al.* (1999)) unit and the two datively-bonded triphenylphosphino ligands. The coordination geometry around copper(I) is best described as a distorted tetrahedron that resembles other complexes including (hfac)Cu(1,5-COD), see: Pampaloni *et al.* (2005); Doyle *et al.* (1985), or (Me_3_P)_2_Cu(acac), see: Shin *et al.* (1991); Chi *et al.* (1992). The bond distances Cu–P, Cu–O, Fe–D1 (1.6401 (9) Å) and Fe–D2 (1.6466 (9) Å) (D1 = centroid of C1 - C5; D2 = centroid of C6 - C10) as well as the bond angles P2–Cu1–P1, P2–Cu1–O1, P2–Cu1–O2, P1–Cu1–O1, P1–Cu1–O2, O2–Cu1–O1 are similar to those ones found for other copper(I) β-diketonate derivatives, see, for example: Yang *et al.* (2001); Marchetti *et al.* (2000); Croxtall *et al.* (2003); Herberhold *et al.* (2004). The atoms Cu1, O1, O2, and C11 - C13 are, as expected, in plane bound (r. m. s. d. of a calculated mean plane = 0.024 Å).

### Experimental

The title complex was synthesized by the consecutive reaction of 100 mg (0.31 mmol) of 1-ferrocenyl-4,4,4-trifluorobutane-1,3-dione (see: du Plessis *et al.* (1999)) with 40 mg (0.36 mmol) of potassium *t*-butanolate (diethyl ether, 298 K, 4 h) followed by addition of 180 mg (0.29 mmol) of [(Ph_3_P)_2_CuCl] (diethyl ether, 298 K, 12 h). After filtration of the reaction mixture through a pad of Celite and removal of all volatile materials in oil-pump vacuum gave the title complex as an orange solid in a yield of 120 mg (0.13 mmol, 45%) based on [(Ph_3_P)_2_CuCl]). This complex is stable in air and light over a period of days. It nicely dissolves in common organic solvents.

### Refinement

*R*(*F^2^* > 2 s(*F^2^*)) = 0.0204 *wR*(*F^2^*) = 0.0501 *S* = 1.028 8248 reflections 532 parameters 1 restraints D*r*_max_ = 0.37 e Å^-3^ D*r*_min_ = -0.25 e Å^-3^

All H atoms were placed in calculated positions and constrained to ride on their parent atoms, with C—H distances of 0.93 Å and *U*_iso_(H) = 1.2*U*_eq_(C).

Atom F2 is claimed to have a suspicious ADP max/min ratio, indicating further disorder. It is checked, that the introduction of a disorder will give non-reasonable occupation factors of *ca* 98 percent to *ca* 2 percent. Furthermore, *U*_eq_ of C14 are claimed. C14 is the pivot atom of a CF_3_ group. PLAT242 ALERT 2 C does already indicate, that false alarms occur for terminal groups such as the *tert*-butyl moiety. As a CF_3_ group is structurally related to a *tert*-butyl moiety we account this error message as a false alarm.

### Crystal data

**Table:**

[CuFe(C_5_H_5_)(C_9_H_5_F_3_O_2_)(C_18_H_15_P)_2_]	*F*(000) = 936
*M**_r_* = 911.15	*D*_x_ = 1.442 Mg m^−^^3^
Monoclinic, *P*2_1_	Mo *K*α radiation, λ = 0.71073 Å
Hall symbol: P 2yb	Cell parameters from 21328 reflections
*a* = 11.02755 (13) Å	θ = 3.0–26.0°
*b* = 16.8178 (2) Å	µ = 0.98 mm^−^^1^
*c* = 12.50787 (14) Å	*T* = 120 K
β = 115.2456 (14)°	Plate, red
*V* = 2098.15 (5) Å^3^	0.56 × 0.48 × 0.18 mm
*Z* = 2	

### Data collection

**Table:**

Oxford Diffraction Gemini S diffractometer	7761 reflections with *I* > 2σ(*I*)
Radiation source: fine-focus sealed tube	*R*_int_ = 0.015
graphite	θ_max_ = 26.1°, θ_min_ = 3.0°
ω scans	*h* = −13→13
Absorption correction: multi-scan (*CrysAlis RED*; Oxford Diffraction, 2006)	*k* = −20→20
*T*_min_ = 0.943, *T*_max_ = 1.000	*l* = −15→15
21096 measured reflections	2 standard reflections every 50 reflections
8248 independent reflections	intensity decay: none

### Refinement

**Table:**

Refinement on *F*^2^	Secondary atom site location: difference Fourier map
Least-squares matrix: full	Hydrogen site location: inferred from neighbouring sites
*R*[*F*^2^ > 2σ(*F*^2^)] = 0.020	H-atom parameters constrained
*wR*(*F*^2^) = 0.052	*w* = 1/[σ^2^(*F*_o_^2^) + (0.0348*P*)^2^ + 0.2178*P*] where *P* = (*F*_o_^2^ + 2*F*_c_^2^)/3
*S* = 1.03	(Δ/σ)_max_ = 0.001
8248 reflections	Δρ_max_ = 0.37 e Å^−^^3^
532 parameters	Δρ_min_ = −0.25 e Å^−^^3^
1 restraint	Absolute structure: Flack (1983), 3945 Friedel pairs
Primary atom site location: structure-invariant direct methods	Flack parameter: −0.010 (6)

### Special details

**Table:**

Experimental. CrysAlis RED, Oxford Diffraction Ltd., Version 1.171.31.7 (release 18-10-2006 CrysAlis171 .NET) (compiled Oct 18 2006,16:28:17) Empirical absorption correction using spherical harmonics, implemented in SCALE3 ABSPACK scaling algorithm.
Geometry. All e.s.d.'s (except the e.s.d. in the dihedral angle between two l.s. planes) are estimated using the full covariance matrix. The cell e.s.d.'s are taken into account individually in the estimation of e.s.d.'s in distances, angles and torsion angles; correlations between e.s.d.'s in cell parameters are only used when they are defined by crystal symmetry. An approximate (isotropic) treatment of cell e.s.d.'s is used for estimating e.s.d.'s involving l.s. planes.
Refinement. Refinement of *F*^2^ against ALL reflections. The weighted *R*-factor *wR* and goodness of fit *S* are based on *F*^2^, conventional *R*-factors *R* are based on *F*, with *F* set to zero for negative *F*^2^. The threshold expression of *F*^2^ > σ(*F*^2^) is used only for calculating *R*-factors(gt) *etc*. and is not relevant to the choice of reflections for refinement. *R*-factors based on *F*^2^ are statistically about twice as large as those based on *F*, and *R*- factors based on ALL data will be even larger.

### Fractional atomic coordinates and isotropic or equivalent isotropic displacement parameters (Å^2^)

**Table:**

	*x*	*y*	*z*	*U*_iso_*/*U*_eq_	
Fe1	0.50411 (2)	0.687238 (16)	0.77772 (2)	0.01723 (6)	
C1	0.55309 (18)	0.61747 (11)	0.67027 (16)	0.0192 (4)	
C2	0.41629 (18)	0.64086 (12)	0.61092 (17)	0.0224 (4)	
H2	0.3778	0.6690	0.5403	0.027*	
C3	0.3491 (2)	0.61353 (13)	0.67863 (19)	0.0292 (5)	
H3	0.2589	0.6213	0.6605	0.035*	
C4	0.4422 (2)	0.57250 (12)	0.7782 (2)	0.0298 (5)	
H4	0.4234	0.5481	0.8361	0.036*	
C5	0.5693 (2)	0.57469 (11)	0.77517 (18)	0.0239 (4)	
H5	0.6485	0.5526	0.8307	0.029*	
C6	0.6210 (2)	0.78630 (13)	0.8155 (2)	0.0320 (5)	
H6	0.6854	0.7976	0.7882	0.038*	
C7	0.4845 (2)	0.80751 (12)	0.75945 (19)	0.0319 (5)	
H7	0.4426	0.8350	0.6885	0.038*	
C8	0.4232 (2)	0.77953 (13)	0.8301 (2)	0.0368 (5)	
H8	0.3332	0.7855	0.8140	0.044*	
C9	0.5199 (3)	0.74116 (13)	0.9286 (2)	0.0412 (6)	
H9	0.5055	0.7172	0.9892	0.049*	
C10	0.6428 (2)	0.74513 (13)	0.92001 (19)	0.0381 (6)	
H10	0.7241	0.7243	0.9738	0.046*	
C11	0.65726 (18)	0.63909 (10)	0.63003 (16)	0.0173 (4)	
C12	0.79289 (18)	0.64258 (12)	0.71616 (15)	0.0194 (4)	
H12	0.8101	0.6281	0.7931	0.023*	
O1	0.61771 (12)	0.65478 (7)	0.52235 (10)	0.0180 (3)	
C13	0.90114 (17)	0.66556 (10)	0.69532 (15)	0.0164 (4)	
O2	0.90944 (10)	0.68040 (9)	0.59961 (9)	0.0173 (2)	
C14	1.03239 (17)	0.68183 (14)	0.80476 (14)	0.0239 (4)	
F1	1.05023 (15)	0.76028 (9)	0.82422 (14)	0.0599 (5)	
F2	1.03981 (12)	0.64954 (12)	0.90381 (11)	0.0592 (5)	
F3	1.13983 (10)	0.65709 (8)	0.79243 (10)	0.0322 (3)	
Cu1	0.739975 (18)	0.684267 (12)	0.439354 (15)	0.01426 (5)	
P1	0.68543 (4)	0.81153 (3)	0.38321 (4)	0.01503 (9)	
C15	0.69285 (18)	0.87771 (11)	0.50199 (15)	0.0167 (4)	
C16	0.79584 (19)	0.86499 (11)	0.61432 (16)	0.0217 (4)	
H16	0.8577	0.8246	0.6258	0.026*	
C17	0.8066 (2)	0.91201 (11)	0.70884 (17)	0.0258 (4)	
H17	0.8761	0.9036	0.7832	0.031*	
C18	0.7137 (2)	0.97155 (12)	0.69227 (17)	0.0239 (4)	
H18	0.7194	1.0023	0.7561	0.029*	
C19	0.6127 (2)	0.98539 (12)	0.58141 (18)	0.0262 (4)	
H19	0.5513	1.0260	0.5705	0.031*	
C20	0.60201 (19)	0.93914 (11)	0.48613 (17)	0.0227 (4)	
H20	0.5341	0.9491	0.4115	0.027*	
C21	0.51264 (17)	0.82467 (10)	0.27141 (15)	0.0175 (4)	
C22	0.41049 (18)	0.79132 (11)	0.29527 (16)	0.0217 (4)	
H22	0.4317	0.7645	0.3661	0.026*	
C23	0.27808 (19)	0.79811 (12)	0.21380 (18)	0.0271 (4)	
H23	0.2103	0.7767	0.2305	0.033*	
C24	0.2460 (2)	0.83704 (13)	0.10670 (18)	0.0319 (5)	
H24	0.1570	0.8413	0.0518	0.038*	
C25	0.3461 (2)	0.86903 (14)	0.08236 (18)	0.0315 (5)	
H25	0.3248	0.8945	0.0106	0.038*	
C26	0.4793 (2)	0.86349 (12)	0.16467 (16)	0.0233 (4)	
H26	0.5464	0.8860	0.1480	0.028*	
C27	0.78703 (17)	0.86077 (11)	0.31957 (15)	0.0171 (4)	
C28	0.83982 (19)	0.93685 (11)	0.35301 (18)	0.0205 (4)	
H28	0.8207	0.9655	0.4076	0.025*	
C29	0.92108 (19)	0.97008 (12)	0.30491 (18)	0.0270 (4)	
H29	0.9576	1.0204	0.3286	0.032*	
C30	0.9476 (2)	0.92845 (14)	0.22210 (19)	0.0299 (5)	
H30	1.0011	0.9510	0.1895	0.036*	
C31	0.8945 (2)	0.85300 (14)	0.18751 (18)	0.0291 (5)	
H31	0.9116	0.8253	0.1311	0.035*	
C32	0.81588 (19)	0.81881 (12)	0.23713 (16)	0.0235 (4)	
H32	0.7823	0.7677	0.2153	0.028*	
P2	0.74219 (4)	0.58095 (3)	0.32711 (4)	0.01338 (9)	
C33	0.58003 (17)	0.56047 (10)	0.20405 (14)	0.0152 (3)	
C34	0.48442 (18)	0.62093 (11)	0.16773 (16)	0.0189 (4)	
H34	0.5054	0.6701	0.2052	0.023*	
C35	0.35846 (19)	0.60869 (12)	0.07654 (17)	0.0226 (4)	
H35	0.2962	0.6498	0.0521	0.027*	
C36	0.32545 (18)	0.53494 (12)	0.02181 (16)	0.0211 (4)	
H36	0.2409	0.5264	−0.0391	0.025*	
C37	0.41859 (19)	0.47433 (11)	0.05821 (16)	0.0214 (4)	
H37	0.3963	0.4248	0.0219	0.026*	
C38	0.54539 (19)	0.48666 (11)	0.14861 (16)	0.0185 (4)	
H38	0.6075	0.4454	0.1723	0.022*	
C39	0.85824 (18)	0.59304 (10)	0.25901 (16)	0.0157 (3)	
C40	0.82338 (19)	0.58266 (12)	0.14010 (16)	0.0235 (4)	
H40	0.7365	0.5674	0.0898	0.028*	
C41	0.9178 (2)	0.59497 (14)	0.09486 (18)	0.0300 (5)	
H41	0.8939	0.5876	0.0147	0.036*	
C42	1.0463 (2)	0.61803 (13)	0.16866 (18)	0.0275 (4)	
H42	1.1091	0.6263	0.1384	0.033*	
C43	1.08173 (19)	0.62878 (12)	0.28778 (18)	0.0256 (4)	
H43	1.1688	0.6439	0.3378	0.031*	
C44	0.98851 (18)	0.61721 (12)	0.33301 (17)	0.0228 (4)	
H44	1.0126	0.6256	0.4130	0.027*	
C45	0.78856 (17)	0.48377 (10)	0.39829 (15)	0.0164 (4)	
C46	0.86044 (19)	0.42620 (12)	0.36963 (18)	0.0214 (4)	
H46	0.8928	0.4373	0.3137	0.026*	
C47	0.88416 (19)	0.35203 (12)	0.42434 (18)	0.0257 (4)	
H47	0.9331	0.3140	0.4054	0.031*	
C48	0.8353 (2)	0.33470 (12)	0.50661 (17)	0.0275 (4)	
H48	0.8503	0.2849	0.5421	0.033*	
C49	0.7642 (2)	0.39143 (13)	0.53613 (17)	0.0267 (4)	
H49	0.7315	0.3797	0.5917	0.032*	
C50	0.74123 (19)	0.46598 (12)	0.48307 (17)	0.0220 (4)	
H50	0.6943	0.5042	0.5040	0.026*	

### Atomic displacement parameters (Å^2^)

**Table:**

	*U*^11^	*U*^22^	*U*^33^	*U*^12^	*U*^13^	*U*^23^
Fe1	0.02191 (12)	0.01476 (11)	0.01945 (12)	−0.00333 (12)	0.01308 (10)	−0.00184 (12)
C1	0.0255 (10)	0.0168 (9)	0.0188 (9)	−0.0054 (8)	0.0129 (8)	−0.0051 (7)
C2	0.0197 (9)	0.0266 (10)	0.0221 (9)	−0.0084 (8)	0.0100 (8)	−0.0086 (8)
C3	0.0273 (10)	0.0311 (11)	0.0379 (11)	−0.0130 (9)	0.0223 (9)	−0.0137 (9)
C4	0.0483 (13)	0.0197 (10)	0.0386 (12)	−0.0148 (9)	0.0350 (11)	−0.0089 (9)
C5	0.0379 (12)	0.0140 (9)	0.0288 (11)	−0.0015 (8)	0.0230 (9)	−0.0014 (8)
C6	0.0369 (12)	0.0237 (11)	0.0456 (13)	−0.0142 (9)	0.0274 (11)	−0.0147 (10)
C7	0.0493 (14)	0.0142 (9)	0.0289 (10)	0.0011 (9)	0.0134 (10)	−0.0002 (9)
C8	0.0300 (11)	0.0281 (11)	0.0588 (15)	−0.0044 (10)	0.0251 (11)	−0.0213 (11)
C9	0.086 (2)	0.0218 (10)	0.0316 (12)	−0.0108 (12)	0.0404 (13)	−0.0089 (9)
C10	0.0408 (13)	0.0249 (11)	0.0297 (12)	0.0070 (10)	−0.0031 (10)	−0.0088 (9)
C11	0.0220 (9)	0.0128 (8)	0.0200 (9)	0.0015 (7)	0.0118 (7)	−0.0024 (7)
C12	0.0203 (9)	0.0255 (10)	0.0147 (8)	0.0031 (8)	0.0096 (7)	0.0022 (7)
O1	0.0179 (6)	0.0223 (6)	0.0155 (6)	−0.0020 (5)	0.0087 (5)	−0.0001 (5)
C13	0.0177 (8)	0.0159 (9)	0.0137 (8)	0.0049 (6)	0.0049 (7)	0.0002 (6)
O2	0.0148 (5)	0.0233 (6)	0.0141 (5)	−0.0004 (6)	0.0063 (4)	−0.0002 (6)
C14	0.0196 (8)	0.0339 (10)	0.0171 (8)	0.0068 (10)	0.0070 (7)	−0.0007 (10)
F1	0.0446 (8)	0.0393 (8)	0.0544 (9)	0.0092 (6)	−0.0186 (7)	−0.0251 (7)
F2	0.0206 (6)	0.1344 (16)	0.0184 (6)	−0.0019 (7)	0.0043 (5)	0.0227 (8)
F3	0.0146 (5)	0.0511 (8)	0.0286 (6)	0.0020 (5)	0.0072 (5)	−0.0039 (5)
Cu1	0.01684 (9)	0.01395 (9)	0.01240 (9)	0.00004 (10)	0.00663 (7)	0.00008 (9)
P1	0.0172 (2)	0.0135 (2)	0.0147 (2)	0.00031 (17)	0.00705 (17)	0.00035 (17)
C15	0.0208 (9)	0.0144 (8)	0.0174 (8)	−0.0036 (7)	0.0105 (7)	−0.0013 (7)
C16	0.0250 (10)	0.0164 (9)	0.0222 (9)	0.0014 (8)	0.0089 (8)	−0.0002 (7)
C17	0.0306 (11)	0.0240 (10)	0.0184 (9)	−0.0033 (8)	0.0061 (8)	−0.0021 (8)
C18	0.0326 (11)	0.0210 (10)	0.0246 (10)	−0.0077 (8)	0.0183 (9)	−0.0074 (8)
C19	0.0249 (10)	0.0233 (10)	0.0339 (11)	−0.0002 (8)	0.0159 (9)	−0.0051 (9)
C20	0.0232 (10)	0.0199 (9)	0.0228 (9)	0.0014 (8)	0.0076 (8)	−0.0023 (8)
C21	0.0198 (9)	0.0137 (9)	0.0186 (9)	−0.0002 (7)	0.0078 (7)	−0.0055 (7)
C22	0.0242 (9)	0.0198 (9)	0.0225 (9)	−0.0010 (8)	0.0114 (8)	−0.0044 (8)
C23	0.0210 (9)	0.0272 (11)	0.0352 (11)	−0.0054 (8)	0.0139 (8)	−0.0116 (9)
C24	0.0208 (10)	0.0375 (12)	0.0290 (11)	0.0029 (9)	0.0027 (8)	−0.0125 (9)
C25	0.0305 (11)	0.0378 (12)	0.0206 (10)	0.0051 (9)	0.0055 (9)	0.0014 (9)
C26	0.0246 (10)	0.0237 (10)	0.0215 (9)	−0.0001 (8)	0.0096 (8)	−0.0009 (8)
C27	0.0155 (9)	0.0185 (9)	0.0164 (8)	0.0011 (7)	0.0058 (7)	0.0043 (7)
C28	0.0195 (9)	0.0165 (9)	0.0231 (10)	0.0041 (8)	0.0068 (8)	0.0039 (8)
C29	0.0204 (10)	0.0233 (10)	0.0335 (11)	−0.0016 (8)	0.0079 (9)	0.0099 (9)
C30	0.0214 (10)	0.0374 (12)	0.0315 (11)	0.0021 (9)	0.0119 (9)	0.0152 (9)
C31	0.0265 (10)	0.0403 (12)	0.0243 (10)	0.0003 (9)	0.0145 (8)	0.0010 (9)
C32	0.0239 (10)	0.0228 (10)	0.0224 (9)	−0.0025 (8)	0.0087 (8)	−0.0002 (8)
P2	0.0143 (2)	0.0134 (2)	0.0123 (2)	0.00083 (17)	0.00556 (17)	0.00071 (16)
C33	0.0160 (8)	0.0185 (9)	0.0125 (8)	−0.0003 (7)	0.0075 (7)	0.0025 (7)
C34	0.0202 (9)	0.0158 (9)	0.0197 (9)	0.0010 (7)	0.0074 (7)	−0.0017 (7)
C35	0.0202 (9)	0.0232 (10)	0.0221 (9)	0.0045 (8)	0.0068 (8)	0.0030 (8)
C36	0.0181 (9)	0.0267 (10)	0.0153 (8)	−0.0060 (8)	0.0041 (7)	−0.0007 (8)
C37	0.0274 (10)	0.0184 (9)	0.0187 (9)	−0.0068 (8)	0.0101 (8)	−0.0049 (7)
C38	0.0215 (9)	0.0152 (9)	0.0179 (9)	0.0022 (7)	0.0074 (7)	0.0016 (7)
C39	0.0184 (9)	0.0111 (8)	0.0189 (9)	0.0029 (7)	0.0092 (7)	0.0025 (7)
C40	0.0162 (9)	0.0333 (11)	0.0201 (9)	−0.0010 (8)	0.0067 (7)	0.0009 (8)
C41	0.0295 (11)	0.0450 (13)	0.0189 (9)	0.0028 (9)	0.0135 (8)	0.0057 (9)
C42	0.0226 (10)	0.0335 (11)	0.0332 (11)	0.0029 (9)	0.0186 (9)	0.0072 (9)
C43	0.0178 (9)	0.0292 (10)	0.0299 (10)	−0.0035 (8)	0.0102 (8)	−0.0017 (9)
C44	0.0209 (9)	0.0260 (10)	0.0212 (9)	−0.0019 (8)	0.0085 (8)	−0.0029 (8)
C45	0.0138 (8)	0.0158 (8)	0.0164 (8)	−0.0008 (7)	0.0034 (7)	0.0015 (7)
C46	0.0192 (9)	0.0192 (9)	0.0246 (10)	0.0013 (8)	0.0082 (8)	0.0012 (8)
C47	0.0209 (10)	0.0188 (9)	0.0318 (10)	0.0053 (7)	0.0058 (8)	0.0021 (8)
C48	0.0241 (10)	0.0173 (9)	0.0288 (10)	−0.0028 (8)	−0.0005 (8)	0.0085 (8)
C49	0.0284 (11)	0.0263 (10)	0.0253 (10)	−0.0034 (9)	0.0114 (9)	0.0092 (8)
C50	0.0229 (10)	0.0217 (10)	0.0210 (9)	−0.0002 (8)	0.0091 (8)	0.0033 (8)

### Geometric parameters (Å, °)

**Table:**

Fe1—C1	2.0233 (18)	C21—C22	1.400 (3)
Fe1—C5	2.0300 (19)	C22—C23	1.384 (3)
Fe1—C10	2.033 (2)	C22—H22	0.9300
Fe1—C8	2.033 (2)	C23—C24	1.394 (3)
Fe1—C9	2.034 (2)	C23—H23	0.9300
Fe1—C6	2.035 (2)	C24—C25	1.374 (3)
Fe1—C7	2.037 (2)	C24—H24	0.9300
Fe1—C2	2.0434 (18)	C25—C26	1.391 (3)
Fe1—C3	2.047 (2)	C25—H25	0.9300
Fe1—C4	2.048 (2)	C26—H26	0.9300
C1—C2	1.423 (3)	C27—C32	1.394 (3)
C1—C5	1.439 (3)	C27—C28	1.394 (3)
C1—C11	1.483 (3)	C28—C29	1.392 (3)
C2—C3	1.419 (3)	C28—H28	0.9300
C2—H2	0.9300	C29—C30	1.381 (3)
C3—C4	1.411 (3)	C29—H29	0.9300
C3—H3	0.9300	C30—C31	1.387 (3)
C4—C5	1.419 (3)	C30—H30	0.9300
C4—H4	0.9300	C31—C32	1.388 (3)
C5—H5	0.9300	C31—H31	0.9300
C6—C10	1.406 (3)	C32—H32	0.9300
C6—C7	1.409 (3)	P2—C39	1.8261 (18)
C6—H6	0.9300	P2—C45	1.8266 (18)
C7—C8	1.403 (3)	P2—C33	1.8270 (17)
C7—H7	0.9300	C33—C38	1.394 (3)
C8—C9	1.397 (4)	C33—C34	1.394 (2)
C8—H8	0.9300	C34—C35	1.386 (3)
C9—C10	1.407 (3)	C34—H34	0.9300
C9—H9	0.9300	C35—C36	1.388 (3)
C10—H10	0.9300	C35—H35	0.9300
C11—O1	1.254 (2)	C36—C37	1.379 (3)
C11—C12	1.424 (3)	C36—H36	0.9300
C12—C13	1.381 (3)	C37—C38	1.389 (3)
C12—H12	0.9300	C37—H37	0.9300
O1—Cu1	2.0821 (12)	C38—H38	0.9300
C13—O2	1.264 (2)	C39—C40	1.379 (3)
C13—C14	1.534 (2)	C39—C44	1.397 (3)
O2—Cu1	2.0783 (11)	C40—C41	1.397 (3)
C14—F2	1.323 (2)	C40—H40	0.9300
C14—F3	1.325 (2)	C41—C42	1.376 (3)
C14—F1	1.341 (3)	C41—H41	0.9300
Cu1—P2	2.2405 (5)	C42—C43	1.382 (3)
Cu1—P1	2.2529 (5)	C42—H42	0.9300
P1—C27	1.8264 (18)	C43—C44	1.382 (3)
P1—C15	1.8300 (17)	C43—H43	0.9300
P1—C21	1.8336 (18)	C44—H44	0.9300
C15—C20	1.393 (3)	C45—C46	1.392 (3)
C15—C16	1.396 (3)	C45—C50	1.400 (3)
C16—C17	1.385 (3)	C46—C47	1.393 (3)
C16—H16	0.9300	C46—H46	0.9300
C17—C18	1.384 (3)	C47—C48	1.381 (3)
C17—H17	0.9300	C47—H47	0.9300
C18—C19	1.378 (3)	C48—C49	1.382 (3)
C18—H18	0.9300	C48—H48	0.9300
C19—C20	1.385 (3)	C49—C50	1.390 (3)
C19—H19	0.9300	C49—H49	0.9300
C20—H20	0.9300	C50—H50	0.9300
C21—C26	1.387 (3)		
			
C1—Fe1—C5	41.59 (8)	O1—Cu1—P2	108.07 (4)
C1—Fe1—C10	123.01 (9)	O2—Cu1—P1	109.94 (4)
C5—Fe1—C10	108.72 (9)	O1—Cu1—P1	103.77 (4)
C1—Fe1—C8	159.22 (9)	P2—Cu1—P1	127.900 (17)
C5—Fe1—C8	157.54 (9)	C27—P1—C15	104.76 (8)
C10—Fe1—C8	67.80 (9)	C27—P1—C21	104.13 (8)
C1—Fe1—C9	159.03 (10)	C15—P1—C21	102.59 (8)
C5—Fe1—C9	122.67 (9)	C27—P1—Cu1	115.77 (6)
C10—Fe1—C9	40.48 (10)	C15—P1—Cu1	114.28 (6)
C8—Fe1—C9	40.18 (10)	C21—P1—Cu1	113.87 (6)
C1—Fe1—C6	107.89 (8)	C20—C15—C16	118.94 (16)
C5—Fe1—C6	125.00 (9)	C20—C15—P1	123.71 (14)
C10—Fe1—C6	40.44 (9)	C16—C15—P1	117.35 (14)
C8—Fe1—C6	67.76 (9)	C17—C16—C15	120.58 (18)
C9—Fe1—C6	67.93 (9)	C17—C16—H16	119.7
C1—Fe1—C7	123.17 (8)	C15—C16—H16	119.7
C5—Fe1—C7	161.03 (9)	C18—C17—C16	119.79 (18)
C10—Fe1—C7	68.09 (9)	C18—C17—H17	120.1
C8—Fe1—C7	40.33 (9)	C16—C17—H17	120.1
C9—Fe1—C7	67.92 (9)	C19—C18—C17	120.14 (17)
C6—Fe1—C7	40.49 (9)	C19—C18—H18	119.9
C1—Fe1—C2	40.97 (8)	C17—C18—H18	119.9
C5—Fe1—C2	69.20 (8)	C18—C19—C20	120.40 (18)
C10—Fe1—C2	158.22 (9)	C18—C19—H19	119.8
C8—Fe1—C2	122.57 (9)	C20—C19—H19	119.8
C9—Fe1—C2	158.99 (10)	C19—C20—C15	120.12 (18)
C6—Fe1—C2	121.82 (8)	C19—C20—H20	119.9
C7—Fe1—C2	106.45 (8)	C15—C20—H20	119.9
C1—Fe1—C3	68.73 (8)	C26—C21—C22	119.10 (17)
C5—Fe1—C3	68.65 (9)	C26—C21—P1	123.61 (14)
C10—Fe1—C3	160.49 (10)	C22—C21—P1	117.26 (13)
C8—Fe1—C3	106.82 (9)	C23—C22—C21	120.16 (18)
C9—Fe1—C3	123.42 (9)	C23—C22—H22	119.9
C6—Fe1—C3	156.84 (10)	C21—C22—H22	119.9
C7—Fe1—C3	120.80 (9)	C22—C23—C24	120.12 (19)
C2—Fe1—C3	40.60 (8)	C22—C23—H23	119.9
C1—Fe1—C4	68.84 (8)	C24—C23—H23	119.9
C5—Fe1—C4	40.73 (8)	C25—C24—C23	119.90 (19)
C10—Fe1—C4	125.19 (9)	C25—C24—H24	120.0
C8—Fe1—C4	121.73 (9)	C23—C24—H24	120.0
C9—Fe1—C4	108.32 (9)	C24—C25—C26	120.26 (19)
C6—Fe1—C4	161.77 (10)	C24—C25—H25	119.9
C7—Fe1—C4	156.43 (10)	C26—C25—H25	119.9
C2—Fe1—C4	68.26 (8)	C21—C26—C25	120.45 (18)
C3—Fe1—C4	40.31 (9)	C21—C26—H26	119.8
C2—C1—C5	107.81 (17)	C25—C26—H26	119.8
C2—C1—C11	123.88 (17)	C32—C27—C28	119.27 (17)
C5—C1—C11	128.23 (17)	C32—C27—P1	117.97 (14)
C2—C1—Fe1	70.27 (10)	C28—C27—P1	122.73 (14)
C5—C1—Fe1	69.45 (10)	C29—C28—C27	120.15 (19)
C11—C1—Fe1	123.25 (13)	C29—C28—H28	119.9
C3—C2—C1	107.85 (18)	C27—C28—H28	119.9
C3—C2—Fe1	69.82 (11)	C30—C29—C28	120.12 (19)
C1—C2—Fe1	68.76 (10)	C30—C29—H29	119.9
C3—C2—H2	126.1	C28—C29—H29	119.9
C1—C2—H2	126.1	C29—C30—C31	120.12 (19)
Fe1—C2—H2	126.9	C29—C30—H30	119.9
C4—C3—C2	108.42 (18)	C31—C30—H30	119.9
C4—C3—Fe1	69.89 (12)	C30—C31—C32	120.02 (19)
C2—C3—Fe1	69.58 (11)	C30—C31—H31	120.0
C4—C3—H3	125.8	C32—C31—H31	120.0
C2—C3—H3	125.8	C31—C32—C27	120.29 (18)
Fe1—C3—H3	126.3	C31—C32—H32	119.9
C3—C4—C5	108.64 (18)	C27—C32—H32	119.9
C3—C4—Fe1	69.80 (12)	C39—P2—C45	102.75 (8)
C5—C4—Fe1	68.97 (11)	C39—P2—C33	104.88 (8)
C3—C4—H4	125.7	C45—P2—C33	102.24 (8)
C5—C4—H4	125.7	C39—P2—Cu1	113.77 (6)
Fe1—C4—H4	127.1	C45—P2—Cu1	117.65 (6)
C4—C5—C1	107.27 (19)	C33—P2—Cu1	113.94 (6)
C4—C5—Fe1	70.31 (12)	C38—C33—C34	118.62 (16)
C1—C5—Fe1	68.96 (11)	C38—C33—P2	123.31 (13)
C4—C5—H5	126.4	C34—C33—P2	118.01 (13)
C1—C5—H5	126.4	C35—C34—C33	120.86 (17)
Fe1—C5—H5	125.9	C35—C34—H34	119.6
C10—C6—C7	108.1 (2)	C33—C34—H34	119.6
C10—C6—Fe1	69.72 (12)	C34—C35—C36	119.91 (17)
C7—C6—Fe1	69.83 (12)	C34—C35—H35	120.0
C10—C6—H6	125.9	C36—C35—H35	120.0
C7—C6—H6	125.9	C37—C36—C35	119.72 (17)
Fe1—C6—H6	126.1	C37—C36—H36	120.1
C8—C7—C6	107.5 (2)	C35—C36—H36	120.1
C8—C7—Fe1	69.70 (12)	C36—C37—C38	120.52 (17)
C6—C7—Fe1	69.68 (12)	C36—C37—H37	119.7
C8—C7—H7	126.2	C38—C37—H37	119.7
C6—C7—H7	126.2	C37—C38—C33	120.35 (17)
Fe1—C7—H7	126.0	C37—C38—H38	119.8
C9—C8—C7	108.6 (2)	C33—C38—H38	119.8
C9—C8—Fe1	69.92 (12)	C40—C39—C44	119.09 (17)
C7—C8—Fe1	69.98 (12)	C40—C39—P2	124.09 (14)
C9—C8—H8	125.7	C44—C39—P2	116.78 (13)
C7—C8—H8	125.7	C39—C40—C41	120.34 (18)
Fe1—C8—H8	126.0	C39—C40—H40	119.8
C8—C9—C10	107.97 (19)	C41—C40—H40	119.8
C8—C9—Fe1	69.89 (12)	C42—C41—C40	120.16 (19)
C10—C9—Fe1	69.74 (12)	C42—C41—H41	119.9
C8—C9—H9	126.0	C40—C41—H41	119.9
C10—C9—H9	126.0	C41—C42—C43	119.79 (18)
Fe1—C9—H9	125.9	C41—C42—H42	120.1
C6—C10—C9	107.8 (2)	C43—C42—H42	120.1
C6—C10—Fe1	69.84 (12)	C42—C43—C44	120.36 (18)
C9—C10—Fe1	69.78 (13)	C42—C43—H43	119.8
C6—C10—H10	126.1	C44—C43—H43	119.8
C9—C10—H10	126.1	C43—C44—C39	120.26 (17)
Fe1—C10—H10	125.9	C43—C44—H44	119.9
O1—C11—C12	125.16 (16)	C39—C44—H44	119.9
O1—C11—C1	116.83 (16)	C46—C45—C50	118.94 (17)
C12—C11—C1	117.99 (16)	C46—C45—P2	124.66 (14)
C13—C12—C11	125.61 (16)	C50—C45—P2	116.31 (14)
C13—C12—H12	117.2	C45—C46—C47	120.32 (19)
C11—C12—H12	117.2	C45—C46—H46	119.8
C11—O1—Cu1	125.67 (11)	C47—C46—H46	119.8
O2—C13—C12	130.66 (16)	C48—C47—C46	120.26 (19)
O2—C13—C14	112.84 (15)	C48—C47—H47	119.9
C12—C13—C14	116.35 (15)	C46—C47—H47	119.9
C13—O2—Cu1	121.41 (10)	C47—C48—C49	119.98 (18)
F2—C14—F3	106.19 (16)	C47—C48—H48	120.0
F2—C14—F1	106.75 (17)	C49—C48—H48	120.0
F3—C14—F1	105.12 (17)	C48—C49—C50	120.23 (18)
F2—C14—C13	114.86 (16)	C48—C49—H49	119.9
F3—C14—C13	112.97 (14)	C50—C49—H49	119.9
F1—C14—C13	110.30 (16)	C49—C50—C45	120.25 (19)
O2—Cu1—O1	91.08 (4)	C49—C50—H50	119.9
O2—Cu1—P2	109.54 (4)	C45—C50—H50	119.9
			
C5—Fe1—C1—C2	118.78 (16)	C5—Fe1—C9—C8	−160.41 (13)
C10—Fe1—C1—C2	−160.17 (12)	C10—Fe1—C9—C8	119.03 (19)
C8—Fe1—C1—C2	−43.9 (3)	C6—Fe1—C9—C8	81.21 (14)
C9—Fe1—C1—C2	166.9 (2)	C7—Fe1—C9—C8	37.36 (14)
C6—Fe1—C1—C2	−118.24 (12)	C2—Fe1—C9—C8	−40.9 (3)
C7—Fe1—C1—C2	−76.30 (14)	C3—Fe1—C9—C8	−75.87 (16)
C3—Fe1—C1—C2	37.41 (12)	C4—Fe1—C9—C8	−117.81 (14)
C4—Fe1—C1—C2	80.79 (13)	C1—Fe1—C9—C10	44.6 (3)
C10—Fe1—C1—C5	81.05 (14)	C5—Fe1—C9—C10	80.55 (15)
C8—Fe1—C1—C5	−162.7 (2)	C8—Fe1—C9—C10	−119.03 (19)
C9—Fe1—C1—C5	48.1 (3)	C6—Fe1—C9—C10	−37.82 (14)
C6—Fe1—C1—C5	122.98 (13)	C7—Fe1—C9—C10	−81.67 (14)
C7—Fe1—C1—C5	164.92 (13)	C2—Fe1—C9—C10	−159.9 (2)
C2—Fe1—C1—C5	−118.78 (16)	C3—Fe1—C9—C10	165.10 (14)
C3—Fe1—C1—C5	−81.37 (13)	C4—Fe1—C9—C10	123.16 (14)
C4—Fe1—C1—C5	−37.98 (13)	C7—C6—C10—C9	−0.2 (2)
C5—Fe1—C1—C11	−123.0 (2)	Fe1—C6—C10—C9	−59.70 (15)
C10—Fe1—C1—C11	−41.94 (19)	C7—C6—C10—Fe1	59.50 (14)
C8—Fe1—C1—C11	74.3 (3)	C8—C9—C10—C6	0.1 (2)
C9—Fe1—C1—C11	−74.9 (3)	Fe1—C9—C10—C6	59.74 (15)
C6—Fe1—C1—C11	−0.01 (18)	C8—C9—C10—Fe1	−59.67 (15)
C7—Fe1—C1—C11	41.93 (19)	C1—Fe1—C10—C6	78.60 (15)
C2—Fe1—C1—C11	118.2 (2)	C5—Fe1—C10—C6	122.41 (13)
C3—Fe1—C1—C11	155.64 (18)	C8—Fe1—C10—C6	−81.30 (14)
C4—Fe1—C1—C11	−160.98 (18)	C9—Fe1—C10—C6	−118.83 (19)
C5—C1—C2—C3	0.5 (2)	C7—Fe1—C10—C6	−37.63 (13)
C11—C1—C2—C3	−176.50 (17)	C2—Fe1—C10—C6	41.8 (3)
Fe1—C1—C2—C3	−59.07 (13)	C3—Fe1—C10—C6	−158.8 (2)
C5—C1—C2—Fe1	59.55 (13)	C4—Fe1—C10—C6	164.67 (13)
C11—C1—C2—Fe1	−117.43 (17)	C1—Fe1—C10—C9	−162.57 (13)
C1—Fe1—C2—C3	119.55 (17)	C5—Fe1—C10—C9	−118.75 (14)
C5—Fe1—C2—C3	81.07 (13)	C8—Fe1—C10—C9	37.54 (14)
C10—Fe1—C2—C3	169.6 (2)	C6—Fe1—C10—C9	118.83 (19)
C8—Fe1—C2—C3	−77.43 (15)	C7—Fe1—C10—C9	81.20 (15)
C9—Fe1—C2—C3	−47.4 (3)	C2—Fe1—C10—C9	160.6 (2)
C6—Fe1—C2—C3	−159.83 (13)	C3—Fe1—C10—C9	−40.0 (3)
C7—Fe1—C2—C3	−118.43 (14)	C4—Fe1—C10—C9	−76.49 (16)
C4—Fe1—C2—C3	37.23 (13)	C2—C1—C11—O1	−24.3 (3)
C5—Fe1—C2—C1	−38.49 (11)	C5—C1—C11—O1	159.41 (18)
C10—Fe1—C2—C1	50.1 (3)	Fe1—C1—C11—O1	−111.61 (17)
C8—Fe1—C2—C1	163.02 (12)	C2—C1—C11—C12	154.08 (17)
C9—Fe1—C2—C1	−166.9 (2)	C5—C1—C11—C12	−22.3 (3)
C6—Fe1—C2—C1	80.61 (14)	Fe1—C1—C11—C12	66.7 (2)
C7—Fe1—C2—C1	122.01 (12)	O1—C11—C12—C13	1.7 (3)
C3—Fe1—C2—C1	−119.55 (17)	C1—C11—C12—C13	−176.47 (17)
C4—Fe1—C2—C1	−82.33 (12)	C12—C11—O1—Cu1	3.4 (2)
C1—C2—C3—C4	−0.9 (2)	C1—C11—O1—Cu1	−178.44 (11)
Fe1—C2—C3—C4	−59.28 (14)	C11—C12—C13—O2	−7.8 (3)
C1—C2—C3—Fe1	58.40 (13)	C11—C12—C13—C14	167.41 (18)
C1—Fe1—C3—C4	81.96 (12)	C12—C13—O2—Cu1	6.8 (3)
C5—Fe1—C3—C4	37.16 (12)	C14—C13—O2—Cu1	−168.52 (12)
C10—Fe1—C3—C4	−48.7 (3)	O2—C13—C14—F2	−164.16 (17)
C8—Fe1—C3—C4	−119.53 (14)	C12—C13—C14—F2	19.8 (3)
C9—Fe1—C3—C4	−78.72 (15)	O2—C13—C14—F3	−42.2 (2)
C6—Fe1—C3—C4	167.84 (19)	C12—C13—C14—F3	141.79 (18)
C7—Fe1—C3—C4	−161.20 (13)	O2—C13—C14—F1	75.17 (19)
C2—Fe1—C3—C4	119.70 (17)	C12—C13—C14—F1	−100.89 (19)
C1—Fe1—C3—C2	−37.74 (12)	C13—O2—Cu1—O1	−1.56 (14)
C5—Fe1—C3—C2	−82.54 (13)	C13—O2—Cu1—P2	−111.18 (13)
C10—Fe1—C3—C2	−168.4 (2)	C13—O2—Cu1—P1	103.52 (13)
C8—Fe1—C3—C2	120.76 (13)	C11—O1—Cu1—O2	−3.09 (14)
C9—Fe1—C3—C2	161.58 (13)	C11—O1—Cu1—P2	107.88 (14)
C6—Fe1—C3—C2	48.1 (3)	C11—O1—Cu1—P1	−113.94 (14)
C7—Fe1—C3—C2	79.10 (14)	O2—Cu1—P1—C27	77.72 (7)
C4—Fe1—C3—C2	−119.70 (17)	O1—Cu1—P1—C27	174.00 (7)
C2—C3—C4—C5	0.9 (2)	P2—Cu1—P1—C27	−59.45 (7)
Fe1—C3—C4—C5	−58.15 (14)	O2—Cu1—P1—C15	−44.16 (7)
C2—C3—C4—Fe1	59.09 (14)	O1—Cu1—P1—C15	52.12 (7)
C1—Fe1—C4—C3	−81.66 (12)	P2—Cu1—P1—C15	178.67 (6)
C5—Fe1—C4—C3	−120.42 (17)	O2—Cu1—P1—C21	−161.62 (7)
C10—Fe1—C4—C3	162.11 (13)	O1—Cu1—P1—C21	−65.34 (7)
C8—Fe1—C4—C3	78.29 (15)	P2—Cu1—P1—C21	61.21 (7)
C9—Fe1—C4—C3	120.43 (14)	C27—P1—C15—C20	89.84 (17)
C6—Fe1—C4—C3	−164.6 (2)	C21—P1—C15—C20	−18.67 (18)
C7—Fe1—C4—C3	43.8 (3)	Cu1—P1—C15—C20	−142.42 (14)
C2—Fe1—C4—C3	−37.48 (12)	C27—P1—C15—C16	−90.27 (15)
C1—Fe1—C4—C5	38.77 (12)	C21—P1—C15—C16	161.22 (14)
C10—Fe1—C4—C5	−77.46 (15)	Cu1—P1—C15—C16	37.46 (16)
C8—Fe1—C4—C5	−161.29 (13)	C20—C15—C16—C17	0.8 (3)
C9—Fe1—C4—C5	−119.14 (14)	P1—C15—C16—C17	−179.08 (15)
C6—Fe1—C4—C5	−44.2 (3)	C15—C16—C17—C18	0.8 (3)
C7—Fe1—C4—C5	164.22 (19)	C16—C17—C18—C19	−1.7 (3)
C2—Fe1—C4—C5	82.94 (13)	C17—C18—C19—C20	1.0 (3)
C3—Fe1—C4—C5	120.42 (17)	C18—C19—C20—C15	0.6 (3)
C3—C4—C5—C1	−0.6 (2)	C16—C15—C20—C19	−1.5 (3)
Fe1—C4—C5—C1	−59.30 (13)	P1—C15—C20—C19	178.40 (14)
C3—C4—C5—Fe1	58.66 (14)	C27—P1—C21—C26	0.86 (17)
C2—C1—C5—C4	0.1 (2)	C15—P1—C21—C26	109.86 (16)
C11—C1—C5—C4	176.90 (18)	Cu1—P1—C21—C26	−126.12 (14)
Fe1—C1—C5—C4	60.16 (13)	C27—P1—C21—C22	178.99 (14)
C2—C1—C5—Fe1	−60.06 (13)	C15—P1—C21—C22	−72.02 (15)
C11—C1—C5—Fe1	116.75 (19)	Cu1—P1—C21—C22	52.01 (15)
C1—Fe1—C5—C4	−118.39 (18)	C26—C21—C22—C23	−0.9 (3)
C10—Fe1—C5—C4	122.61 (14)	P1—C21—C22—C23	−179.06 (14)
C8—Fe1—C5—C4	45.6 (3)	C21—C22—C23—C24	1.1 (3)
C9—Fe1—C5—C4	80.06 (16)	C22—C23—C24—C25	−0.3 (3)
C6—Fe1—C5—C4	164.56 (13)	C23—C24—C25—C26	−0.7 (3)
C7—Fe1—C5—C4	−160.5 (2)	C22—C21—C26—C25	−0.1 (3)
C2—Fe1—C5—C4	−80.45 (13)	P1—C21—C26—C25	177.94 (15)
C3—Fe1—C5—C4	−36.80 (13)	C24—C25—C26—C21	0.9 (3)
C10—Fe1—C5—C1	−119.00 (13)	C15—P1—C27—C32	171.14 (14)
C8—Fe1—C5—C1	164.0 (2)	C21—P1—C27—C32	−81.48 (15)
C9—Fe1—C5—C1	−161.55 (12)	Cu1—P1—C27—C32	44.31 (16)
C6—Fe1—C5—C1	−77.05 (15)	C15—P1—C27—C28	−6.69 (17)
C7—Fe1—C5—C1	−42.1 (3)	C21—P1—C27—C28	100.69 (16)
C2—Fe1—C5—C1	37.94 (11)	Cu1—P1—C27—C28	−133.52 (14)
C3—Fe1—C5—C1	81.59 (12)	C32—C27—C28—C29	−0.4 (3)
C4—Fe1—C5—C1	118.39 (18)	P1—C27—C28—C29	177.39 (14)
C1—Fe1—C6—C10	−120.26 (13)	C27—C28—C29—C30	1.3 (3)
C5—Fe1—C6—C10	−77.43 (15)	C28—C29—C30—C31	−0.7 (3)
C8—Fe1—C6—C10	81.40 (15)	C29—C30—C31—C32	−0.7 (3)
C9—Fe1—C6—C10	37.86 (14)	C30—C31—C32—C27	1.6 (3)
C7—Fe1—C6—C10	119.25 (19)	C28—C27—C32—C31	−1.0 (3)
C2—Fe1—C6—C10	−163.09 (13)	P1—C27—C32—C31	−178.94 (15)
C3—Fe1—C6—C10	162.1 (2)	O2—Cu1—P2—C39	−76.44 (7)
C4—Fe1—C6—C10	−43.7 (3)	O1—Cu1—P2—C39	−174.30 (7)
C1—Fe1—C6—C7	120.49 (13)	P1—Cu1—P2—C39	60.86 (7)
C5—Fe1—C6—C7	163.32 (12)	O2—Cu1—P2—C45	43.76 (8)
C10—Fe1—C6—C7	−119.25 (19)	O1—Cu1—P2—C45	−54.10 (7)
C8—Fe1—C6—C7	−37.85 (13)	P1—Cu1—P2—C45	−178.94 (6)
C9—Fe1—C6—C7	−81.39 (15)	O2—Cu1—P2—C33	163.39 (7)
C2—Fe1—C6—C7	77.66 (14)	O1—Cu1—P2—C33	65.53 (7)
C3—Fe1—C6—C7	42.9 (3)	P1—Cu1—P2—C33	−59.31 (6)
C4—Fe1—C6—C7	−162.9 (2)	C39—P2—C33—C38	77.40 (16)
C10—C6—C7—C8	0.2 (2)	C45—P2—C33—C38	−29.54 (16)
Fe1—C6—C7—C8	59.68 (15)	Cu1—P2—C33—C38	−157.55 (13)
C10—C6—C7—Fe1	−59.43 (15)	C39—P2—C33—C34	−105.59 (14)
C1—Fe1—C7—C8	162.93 (13)	C45—P2—C33—C34	147.47 (14)
C5—Fe1—C7—C8	−165.0 (2)	Cu1—P2—C33—C34	19.46 (15)
C10—Fe1—C7—C8	−81.05 (15)	C38—C33—C34—C35	−1.5 (3)
C9—Fe1—C7—C8	−37.23 (14)	P2—C33—C34—C35	−178.64 (14)
C6—Fe1—C7—C8	−118.64 (19)	C33—C34—C35—C36	1.3 (3)
C2—Fe1—C7—C8	121.31 (13)	C34—C35—C36—C37	−0.3 (3)
C3—Fe1—C7—C8	79.52 (15)	C35—C36—C37—C38	−0.4 (3)
C4—Fe1—C7—C8	48.1 (3)	C36—C37—C38—C33	0.2 (3)
C1—Fe1—C7—C6	−78.43 (14)	C34—C33—C38—C37	0.7 (3)
C5—Fe1—C7—C6	−46.3 (3)	P2—C33—C38—C37	177.72 (14)
C10—Fe1—C7—C6	37.59 (14)	C45—P2—C39—C40	102.87 (17)
C8—Fe1—C7—C6	118.64 (19)	C33—P2—C39—C40	−3.69 (18)
C9—Fe1—C7—C6	81.40 (15)	Cu1—P2—C39—C40	−128.84 (15)
C2—Fe1—C7—C6	−120.06 (13)	C45—P2—C39—C44	−79.61 (15)
C3—Fe1—C7—C6	−161.84 (12)	C33—P2—C39—C44	173.83 (14)
C4—Fe1—C7—C6	166.74 (19)	Cu1—P2—C39—C44	48.68 (15)
C6—C7—C8—C9	−0.2 (2)	C44—C39—C40—C41	1.0 (3)
Fe1—C7—C8—C9	59.46 (15)	P2—C39—C40—C41	178.47 (16)
C6—C7—C8—Fe1	−59.66 (14)	C39—C40—C41—C42	−0.4 (3)
C1—Fe1—C8—C9	−163.5 (2)	C40—C41—C42—C43	0.2 (3)
C5—Fe1—C8—C9	47.6 (3)	C41—C42—C43—C44	−0.6 (3)
C10—Fe1—C8—C9	−37.81 (14)	C42—C43—C44—C39	1.2 (3)
C6—Fe1—C8—C9	−81.66 (15)	C40—C39—C44—C43	−1.4 (3)
C7—Fe1—C8—C9	−119.66 (19)	P2—C39—C44—C43	−179.08 (15)
C2—Fe1—C8—C9	163.84 (13)	C39—P2—C45—C46	−19.17 (18)
C3—Fe1—C8—C9	122.27 (14)	C33—P2—C45—C46	89.41 (17)
C4—Fe1—C8—C9	80.83 (16)	Cu1—P2—C45—C46	−144.98 (14)
C1—Fe1—C8—C7	−43.8 (3)	C39—P2—C45—C50	164.25 (14)
C5—Fe1—C8—C7	167.3 (2)	C33—P2—C45—C50	−87.17 (15)
C10—Fe1—C8—C7	81.84 (15)	Cu1—P2—C45—C50	38.44 (16)
C9—Fe1—C8—C7	119.66 (19)	C50—C45—C46—C47	0.3 (3)
C6—Fe1—C8—C7	38.00 (13)	P2—C45—C46—C47	−176.24 (15)
C2—Fe1—C8—C7	−76.50 (15)	C45—C46—C47—C48	0.7 (3)
C3—Fe1—C8—C7	−118.07 (14)	C46—C47—C48—C49	−0.9 (3)
C4—Fe1—C8—C7	−159.51 (13)	C47—C48—C49—C50	0.2 (3)
C7—C8—C9—C10	0.1 (2)	C48—C49—C50—C45	0.8 (3)
Fe1—C8—C9—C10	59.58 (15)	C46—C45—C50—C49	−1.0 (3)
C7—C8—C9—Fe1	−59.49 (15)	P2—C45—C50—C49	175.78 (15)
C1—Fe1—C9—C8	163.6 (2)		
